# Diagnostic value of inflammatory indicators for surgical site infection in patients with breast cancer

**DOI:** 10.3389/fcimb.2023.1286313

**Published:** 2023-10-25

**Authors:** Dongmei Li, Shanshan Ding, Jie Li, Xianglu Liao, Kun Ru, Lisheng Liu, Wenjing Shang

**Affiliations:** Department of Clinical Laboratory, Shandong Cancer Hospital and Institute, Shandong First Medical University and Shandong Academy of Medical Sciences, Jinan, Shandong, China

**Keywords:** SSI, inflammatory indicators, *Staphylococcus aureus*, antibiotic resistance, breast cancer, diagnostic value

## Abstract

**Background:**

Breast cancer is the most commonly diagnostic cancer in women worldwide. The main treatment for these patients is surgery. However, there is a high incidence of surgical site infection (SSI) in breast cancer patients. The aim of this study was to identify effective infection-related diagnostic markers for timely diagnosis and treatment of SSI.

**Methods:**

This retrospective study included 263 breast cancer patients who were treated between July 2018 and March 2023 at the Shandong Cancer Hospital and Institute. We analyzed differences between the SSI group and control group and differences before and during infection in the SSI group. Finally, we tested the distribution of pathogenic microorganisms and their susceptibility to antibiotics.

**Results:**

Compared with preoperative inflammatory indicators, white blood cells (WBC), neutrophils (NEU), absolute neutrophil count to the absolute lymphocyte count (NLR), D2 polymers (D-Dimer) and fibrinogen (FIB) were significantly increased, while lymphocytes (LYM), albumin (ALB) and prealbumin (PA) were significantly decreased in the SSI group. Compared with uninfected patients, WBC, NEU, NLR and FIB were significantly increased, ALB and PA were significantly decreased in SSI patients, while LYM and D-Dimer did not differ significantly. The distribution of infection bacteria in SSI patients showed that the proportion of patients with *Staphylococcus aureus* infection was as high as 70.41%; of those patients, 19.33% had methicillin-resistant *Staphylococcus aureus* (MRSA) infection. The area under the curves (AUCs) of the receiver operating curves (ROCs) for WBC, NEU, NLR, FIB, ALB and PA were 0.807, 0.811, 0.730, 0.705, 0.663 and 0.796, respectively. The AUCs for other inflammatory indicators were not statistically significant. There was no significant difference in antibiotic resistance for *Staphylococcus aureus* when compared to that of gram-positive bacteria. The resistance of gram-positive bacteria to ceftriaxone (CRO), cefoxitin (FOX), chloramphenicol (CHL), minocycline (MNO) and tetracycline (TCY) was lower than that of gram-negative bacteria, while the resistance to gentamicin (GEN) was higher.

**Conclusion:**

This study demonstrated that WBC, NEU, NLR, FIB and PA have good predictive value for identifying patients at risk of SSI. The cut-off values of inflammatory indicators can be helpful in the prevention and diagnosis of SSI.

## 1 Introduction

Female breast cancer is the leading cause of global cancer incidence, with an estimated 2.3 million new cases each year, thus representing 11.7% of all new cancer cases (Sung et al., 2021). Effective treatments are critical for the prognosis and survival of patients with breast cancer (Maajani et al., 2019). Mastectomy is regarded as a routine surgical procedure for patients with breast cancer. However, SSI after breast surgery can lead to very poor consequences, such as poor outcomes and delayed access to chemotherapy and radiation therapy (Tejirian et al., 2006; Bernier, 2015). The factors influencing SSI have been classified into patient characteristics and surgical related-process, including age, body mass index (BMI), smoking, diabetes mellitus, preoperative treatment, the size of the resection site, prosthesis implantation, tumor progression and metastasis (El-Tamer et al., 2007; Committee on Gynecological Practice, 2015; Zhang et al., 2020). Moreover, evidence has shown that the five-year risk of breast cancer recurrence may be higher in case of SSI (Savioli et al., 2020; Wu et al., 2022). Therefore, it is necessary to identify rapid and effective inflammatory indicators for diagnosing SSI if we are to manage the treatment of patients with breast cancer in a timely manner.

Blood samples have several advantages for the analysis of biomarkers; for example, the blood can be assessed easily in a manner that is not traumatic for the patient. Therefore, the identification of relevant biomarkers of infection in blood samples represents a particularly exciting focus for research (Li et al., 2023). There are several classic infection-related indicators, particularly are procalcitonin (PCT) and C-reactive-protein (CRP) (Ruan et al., 2018; Stocker et al., 2021). Under normal conditions, thyroid C cells and adipose cells secrete and produce hormonally active PCT (Tan et al., 2021). The plasma levels of PCT are known to be increased in severe bacterial, fungal, and parasitic infections, as well as in sepsis and multiple organ failure (Kyriazopoulou et al., 2021). The levels of CRP are known to increase rapidly in blood plasma during infection or tissue damage (Sheinenzon et al., 2021; Cooper et al., 2023). CRP can activate complement and strengthen phagocytic cells, thus increasing the body’s ability to remove pathogenic microorganisms and damaged, necrotic, apoptosis cells. Thus, CRP plays an important protective role in the natural immune process of the body (Ngwa and Agrawal, 2019). It is also possible that WBC, NEU, and NLR in blood plasma could represent informative biomarkers for patients with infection (Christensen et al., 2013; Russell et al., 2019). In addition, indicators related to blood clotting function have also been closely linked to infection. For example, the levels of D-Dimer in patients with *Mycoplasma pneumoniae* pneumonia are known to increase; furthermore, patients with high levels of D-Dimer had more severe clinical manifestations and required longer duration of treatment (Zheng et al., 2021). Fibrinogen is an acute phase reaction proteins and is known to be elevated during infection (Engelmann and Massberg, 2013). Moreover, nutritional indicators including PA and ALB can play an important role in reducing the risk of SSI and postoperative complications (Zhou et al., 2017; Roche et al., 2018).

The distribution of microflora exhibits unique characteristics in breast cancer patients with SSI. Gram-positive bacteria have a higher rate of infection than Gram-negative bacteria, and *Staphylococcus aureus* has the highest probability of infection (Felippe et al., 2007; O'Connor et al., 2020). Prior to surgery for breast cancer, clinicians routinely prescribe antibiotics to prevent SSI. However, some studies have shown that the preoperative use of antibiotics has no direct benefit in reducing the risk of SSI in breast cancer patients (Zhang et al., 2020; Stallard et al., 2022). Furthermore, the overuse of antibiotics can increase bacterial resistance and leads to infections that are more difficult to treat; this can lead to poor outcome for the patients involved (Segura-Egea et al., 2017). Therefore, diagnosing or predicting SSI early and accurately will significantly improve clinical outcomes and reduce the overuse of antibiotics (Celik et al., 2022).

In this study, we analyzed changes in the levels of inflammatory indicators before and during SSI in a cohort of patients undergoing surgery for breast cancer. In addition, we also compared changes of inflammatory markers in breast cancer patients with surgical infection and those without surgical infection. By performing these analyses, we were able to explore the diagnostic efficacy of inflammatory indicators in SSI. Next, we analyzed different types of bacteria, the changes of inflammatory indicators caused by different bacteria, and the antibiotic resistance of different bacteria. Our overall goal was to provide timely and effective treatment for breast cancer patients with SSI.

## 2 Materials and methods

### 2.1 Participants

We conducted a retrospective study of breast cancer patients who underwent breast surgery between July 2018 and March 2023 at the Shandong Cancer Hospital and Institute. Electronic medical records and the Ruimei Laboratory Information System version 6.0 (rmlis, Huangpu District, Shanghai, China) were used to collect clinical information. SSIs were identified by the Centers for Disease Control and Prevention/National Healthcare Safety Network (CDC/NHSN) surveillance definition of health care-associated infections (Horan et al., 2008). SSI occurred within 30 days after surgery. SSI needs to meet at least one of the following conditions (1): pathogenic microorganisms isolated from cultures (2); purulent drainage from the incision (3); presence of at least one of the following symptoms of infection: localized swelling, heat, pain or erythema (4); diagnosis of SSI by the surgeon. This study involved 466 breast cancer patients. 153 patients were excluded because duplicate or insufficient data. Another 50 patients were excluded due to the infection was not at the surgical site or was not caused by the surgery. Finally, 169 patients were classified into SSI group and 94 patients were classified into control group. SSI was diagnosed on the basis of information from electronic medical records, including clinical symptoms, histopathology, microbiological culture, sensitivity results and medication charts. The studies involving human participants were reviewed and approved by the Ethics Committee of the Shandong Cancer Hospital and Institute. The patients/participants provided their written informed consent to participate in this study.

### 2.2 Analysis of inflammatory indicators

The data used in this study were collected from electronic medical records and the Ruimei Laboratory Information System. An automatic hematological analyzer Sysmex XN9000 (Sysmex Corporation, Kobe, Japan) was utilized to analyze blood cells. The normal reference ranges of WBC, NEU, LYM and NLR were 3.5 - 9.5×10^9^, 1.8 - 6.3×10^9^, 1.1 - 3.2×10^9^ and 1.0 - 2.0, respectively. D-Dimer and FIB was analyzed with an ACL 750 system (Chicago, IL, USA). The reference ranges of D-Dimer and FIB were 0 - 1 mg/L and 2 - 4 g/L, respectively. ALB, PA and hsCRP were analyzed using a Beckman Coulter analyzer AU5800 (Beckman Coulter, CA, USA). The reference ranges of ALB and PA were 40 - 55 g/L and 0.25 - 0.4 g/L, respectively. An hsCRP level < 10 mg/L was considered to indicate no systemic infection. A COBAS E801 immunoassay analyzer (Roche Diagnostics GmbH, Mannheim, Germany) was used to quantify PCT. The levels of PCT should be < 0.05 μg/L in healthy individuals. Blood tests were performed when the patient had signs and symptoms of infection. All the above tests were carried out according to the manufacturer’s instructions.

### 2.3 Culture and identification of microorganisms

Samples used for culture included puncture fluid, drainage fluid, tissue, wound secretion, pus and wound swab. Samples were inoculated on Columbia Blood Agar Plates, Chocolate Agar Plates and MacConkey Agar Plates and a BRUKER microflex MALDI TOF/TOF Mass Spectrometer (BRUKER Corporation, Massachusetts, USA) was used to identify microorganism from cultured samples. A BD Phoenix M50 automated microbial system (Becton, Dickinson and Company, New Jersey, USA) was used to perform antimicrobial susceptibility tests. The procedures were performed in accordance with the manufacturer’s instructions and the BC standards of the Clinical Laboratory Standards Institute.

### 2.4 Statistical analysis

Statistical analyses were performed using SPSS software version 23.0 (SPSS, IL, USA) and GraphPad Prism version 9.0 (GraphPad Software, CA, USA). Comparisons of non-normally distributed data were analyzed by Mann-Whitney U tests. Comparisons of normally distributed data were performed by t tests. ROC curves were used to estimate the diagnostic value of inflammatory indicators. Youden’s index was used to determine cut-off points by optimally balancing sensitivity and specificity. *p* < 0.05 was considered statistically significant.

## 3 Results

### 3.1 Demographics and clinical characteristics of the study population

**Figure 1:**
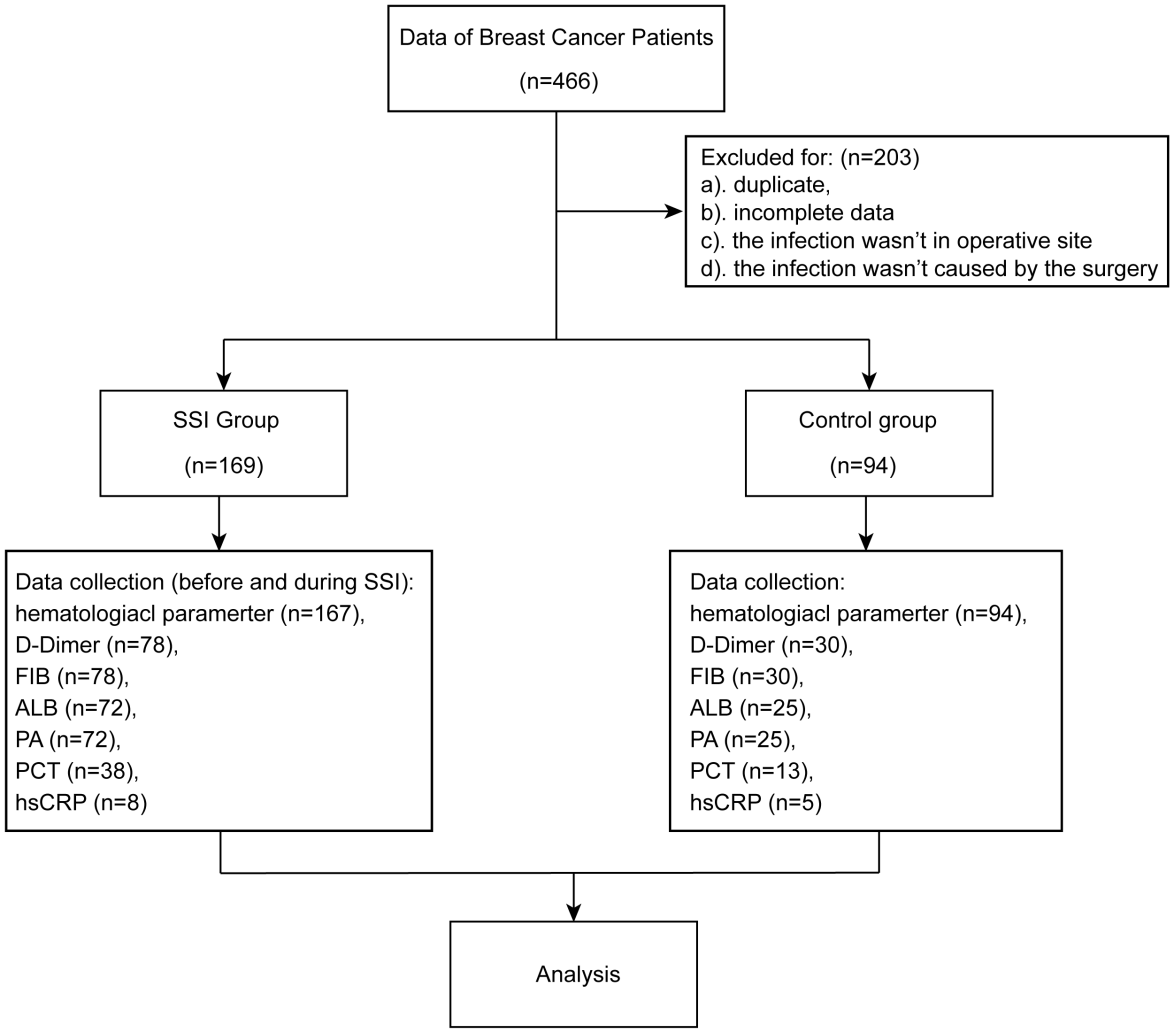
Flow diagram of case collection.

A total of 466 breast cancer patients who underwent breast surgery were included in this study. Of the eligible patients, 169 were classified into SSI group and 94 were classified into control group **Figure 1**. More than 70% of patients were under the age of 60 years. 262 females and 1 male were included in this study. We found that arterial hypertension, diabetes mellitus, preoperative therapy, excision mode, prosthesis implantation, tumor histopathological feature and prophylactic antibiotics were not significantly correlated with SSI. However, we did find that BMI was significantly correlated with SSI. Detailed characteristics of the subjects are shown in **Table 1**.

**Table 1:** Clinical parameters of breast cancer patients.

Clinical parameters	SSI (n=169)	Control group (n=94)	*p*-Value
Age, years, n (%)			0.1376
<60	122 (72.19)	71 (75.53)	
≥60	47 (27.81)	23 (24.47)	
Gender, n (%)			0.1736
Male	1 (0.59)	0 (0)	
Female	168 (99.41)	94 (100)	
Arterial hypertension, n (%)			0.1709
Yes	37 (21.89)	20 (21.28)	
No	132 (78.11)	74 (78.72)	
Body Mass Index, n (%)			0.0141
>25	119 (70.41)	80 (85.11)	
<25	50 (29.59)	14 (14.89)	
Diabetes mellitus			0.1538
Yes	20 (11.83)	8 (8.51)	
No	149 (88.17)	86 (91.49)	
Preoperative therapy			0.1935
Yes	29 (17.16)	18 (19.15)	
No	140 (82.84)	76 (80.85)	
Excision mode			0.1439
Partial excision	19 (11.24)	6 (6.38)	
Total excision	150 (88.76)	88 (93.62)	
Surgery			0.2029
Unilateral surgery	19 (11.24)	14 (14.89)	
Bilateral surgery	150 (88.76)	80 (85.11)	
Prosthesis implantation			0.1863
Yes	10 (5.92)	7 (7.45)	
No	159 (94.08)	87 (92.55)	
Lymphatic metastasis			0.5000
Yes	96 (56.80)	47 (50.00)	
No	73 (43.20)	47 (50.00)	
Carcinoma histology			0.1912
Invasive ductal	147 (86.98)	81 (86.17)	
Non-invasive	18 (10.65)	13 (13.83)	
Invasive lobular	4 (2.37)	0	
ASA Score			0.0720
I or II	136 (80.47)	88 (93.62)	
≥ III	33 (19.53)	6 (6.38)	
Tumor size(cm)			0.3767
≤2	36 (21.30)	37 (39.36)	
2-5	41 (24.26)	20 (21.28)	
>5	92 (54.44)	37 (39.36)	
Prophylactic antibiotics			0.2174
Yes	156 (92.31)	81 (86.17)	
No	13 (7.69)	13 (13.83)	

### 3.2 Changes of inflammatory indicators before and during SSI in the same breast cancer patients

**Figure 2:**
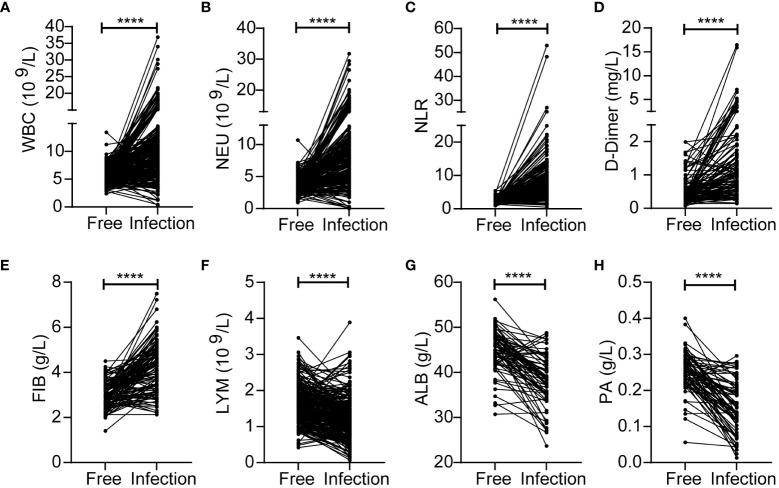
Changes of inflammatory indicators before and during SSI in the breast cancer patients with SSI. The levels of WBC **(A)**, NEU **(B)**, NLR **(C)**, D-Dimer **(D)**, FIB **(E)**, LYM **(F)**, ALB **(G)**, PA **(H)**. ****p < 0.0001.

First, we identified inflammatory indicators associated with SSI by assessing a range of indicators before and during SSI in the same patients with breast cancer. Analysis showed that the levels of WBC, NEU, NLR, D-Dimer and FIB were significantly higher during SSI than in control subjects (**Figures 2A-E**). However, the levels of LYM, ALB and PA were lower during SSI than the non-infected control subjects (**Figures 2F-H**). Detailed information, including the median levels and ranges of inflammatory indicators are presented in **Table 2**.

**Table 2:** Comparison of inflammatory indicators before and during bacterial infection in the same breast cancer patients.

Variable	germ-free	bacteria-infected group	*p*-value
WBC median (range) (10^9^/L)	5.58 (2.38-11.22)	10.30 (0.43-21.65)	<0.0001
NEU median (range) (10^9^/L)	3.42 (0.94-7.19)	8.29 (0.15-20.06)	<0.0001
NLR median (range)	2.07 (0.89-5.53)	6.61 (0.51-52.93)	<0.0001
D-Dimer (range) (mg/L)	0.34 (0.06-1.99)	1.04 (0.14-15.87)	<0.0001
FIB median (range) (g/L)	2.95 (1.4-4.5)	4.15 (2.13-7.49)	<0.0001
LYM median (range) (10^9^/L)	1.61 (0.52-3.46)	1.25 (0.13-3.89)	<0.0001
ALB (range) (g/L)	45.9 (30.7-56.2)	38.7 (23.7-48.8)	<0.0001
PA (range) (g/L)	0.26 (0.06-0.40)	0.15 (0.01-0.30)	<0.0001

### 3.3 Changes of inflammatory indicators in SSI and control groups

**Figure 3:**
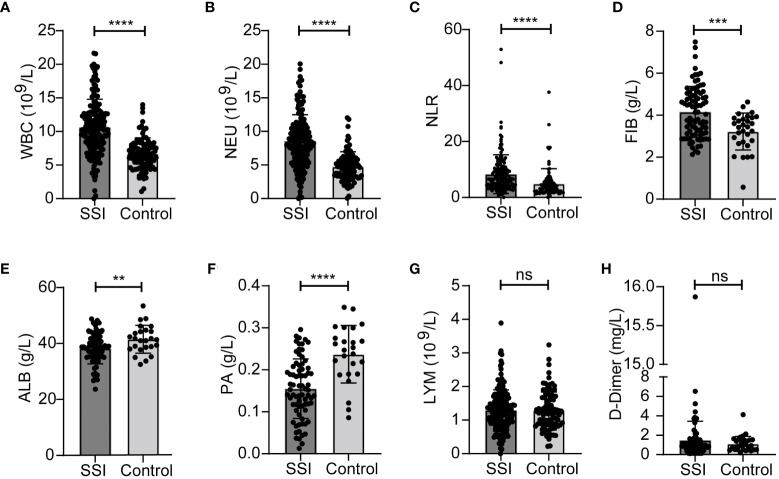
Changes of inflammatory indicators in SSI and control groups. The levels of WBC **(A)**, NEU **(B)**, NLR **(C)**, FIB **(D)**, ALB **(E)**, PA **(F)**, LYM **(G)**, D-Dimer **(H)**. **p < 0.01, ***P < 0.001, ****p < 0.0001. ns, significance.

In order to better explore the changes of inflammatory indicators in breast patients undergoing surgery, we divided patients into SSI group and a postoperative control group according to whether they developed surgery site infection or not. We found that the levels of WBC, NEU, NLR and FIB were significantly higher, while ALB and PA were lower in the SSI group than those in the control group (**Figures 3A-F**). There were no significant between-group differences for the levels of LYM and D-Dimer (**Figures 3G, H**). The median levels and ranges of inflammatory indicators are shown in **Table 3**. Only a small amount of PCT and hsCRP data were available; however, analysis showed that there was no significant difference between the SSI and control groups in terms of PCT and hsCRP (**Figure S1**).

**Table 3:** Comparison of inflammatory indicators in Surgical site infection (SSI) and control group.

Variable	SSI	Control group	*p*-value
WBC median (range) (10^9^/L)	10.30 (0.43-21.65)	6.61 (1.07-13.98)	<0.0001
NEU median (range) (10^9^/L)	8.29 (0.15-20.06)	4.64 (0.06-12.04)	<0.0001
NLR median (range)	6.61 (0.51-52.94)	3.63 (0.08-37.61)	<0.0001
FIB median (range) (g/L)	4.14 (2.13-7.49)	3.44 (0.57-4.63)	0.0002
ALB (range) (g/L)	40.0 (23.7-48.8)	41.4 (32.5-53.4)	0.0097
PA (range) (g/L)	0.15 (0.01-0.30)	0.25 (0.09-0.35)	<0.0001
LYM median (range) (10^9^/L)	1.25 (0.13-3.89)	1.24 (0.22-3.24)	0.9516
D-Dimer (range) (mg/L)	0.99 (0.11-15.87)	0.96 (0.18-4.12)	0.3303

### 3.4 Pathogenic microorganisms and infections

**Figure 4:**
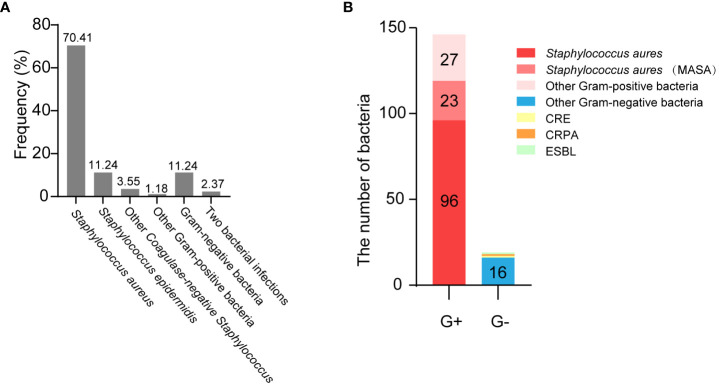
The distribution of pathogenic microorganisms responsible for infected in the breast cancer patients with SSI. **(A)** The frequency of isolates in breast cancer patients with SSI. **(B)** The distribution of muti-drug resistant bacteria.

Next, we analyzed the distribution of pathogenic microorganisms responsible for infected in the breast patients with SSI. Analysis showed that *Staphylococcus aureus* accounted for the highest proportion (up to 70.41%), followed by *Staphylococcus epidermidis* (11.24%); gram-negative bacteria accounted for only 11.24%. In addition, 2.37% of patients had infections caused by two different types of bacteria (**Figure 4A**). Details of bacterial distribution are shown in **Table 4**. Importantly, of the patients infected with *Staphylococcus aureus*, 19.32% had MRSA (**Figure 4B**), thus highlighting the urgent need to develop methods to diagnose and treat infection as early as possible.

**Table 4:** Distribution of infection-associated bacteria.

Microorganism	n (%)
Gram-positive bacteria	146 (86.39)
*Staphylococcus aures*	119 (70.41%)
*Staphylococcus epidermidis*	19 (11.24%)
Other *Coagulase-negative Staphylococcus*	6 (3.55%)
Others	2 (1.18%)
Gram-negative bacteria	19 (11.24%)
*Pseudomonas aeruginosa*	7 (4.14%)
*Escherichia coli*	3 (1.78%)
*Enterobacter cloacae*	2 (1.18%)
*Serratia marcescens*	2 (1.18%)
*Acinetobacter*	2 (1.18%)
Others	3 (1.78%)
Two bacterial infections	4 (2.37%)

**Figure 5:**
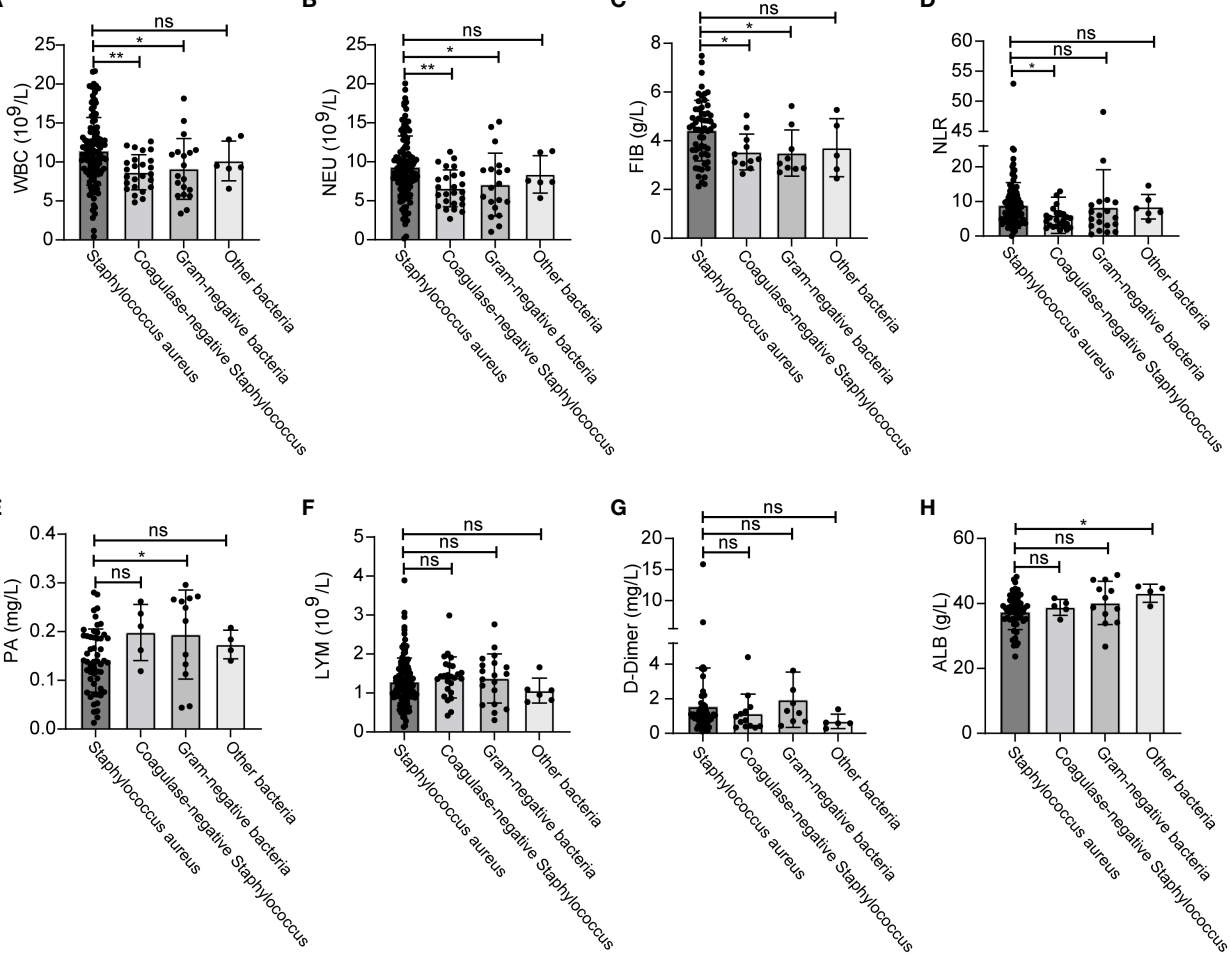
Changes of inflammatory indicators after infection with different bacteria. The levels of WBC **(A)**, NEU **(B)**, FIB **(C)**, NLR **(D)**, PA **(E)**, LYM **(F)**, D-Dimer **(G)**, ALB **(H)**. *p < 0.5, **p < 0.01. ns, significance.

Next, we analyzed the effects of different types of bacterial infections on various inflammatory indicators. The increase of WBC, NEU and FIB after infection with *Staphylococcus aureus* was greater than that of coagulase-negative *Staphylococcus* and gram-negative bacteria (**Figures 5A-C**). The increase of NLR after infection with *Staphylococcus aureus* was greater than that of gram-negative bacteria (**Figure 5D**). In addition, PA levels were significantly higher after infection with gram-negative bacteria than after infection with *Staphylococcus aureus* (**Figure 5E**). Distinct types of bacterial infection had no significant effects on the levels of LMY, D-Dimer and ALB (**Figures 5F-H**). The changes and ranges of various inflammatory indicators between different bacterial infections are shown in **Table 5**. Due to the small amount of data available for PCT and hsCRP, we were unable to identify any significant changes associated with *Staphylococcus aureus* and other bacterial infections (**Figure S2**).

**Table 5:** Comparison of inflammatory indicators between different bacterial infection.

Variable	*Staphylococcus aureus*	Coagulase-negative *Staphylococcus*	Gram-negative bacteria	Other bacteria
WBC median (range) (10^9^/L)	11.0 (0.43-21.65)	8.44 (4.82-12.63)	8.2 (3.39-18.14)	9.52 (6.58-13.35)
NEU median (range) (10^9^/L)	8.89 (0.15-20.06)	6.12 (2.67-11.29)	6.66 (1.00-15.13)	7.58 (5.34-11.36)
FIB median (range) (g/L)	4.52 (2.13-7.49)	3.25 (2.64-5.04)	3.07 (2.71-5.43)	3.40 (2.46-5.27)
NLR median (range)	7.39 (1.15-52.94)	4.68 (1.66-26.88)	5.28 (0.51-48.23)	7.98 (4.46-14.56)
PA median (range) (g/L)	0.14 (0.01-0.28)	0.21 (0.12-0.26)	0.25 (0.04-0.30)	0.18 (0.14-0.21)
LYM median (range) (10^9^/L)	1.41 (0.13-1.55)	1.42 (0.42-2.99)	1.45 (0.30-2.76)	1.07 (0.77-1.66)
D-Dimer (mg/L)	1.04 (0.11-15.87)	0.75 (0.33-4.40)	1.30 (0.44-5.24)	0.59 (0.28-1.39)
ALB (g/L)	37.7 (23.7-48.2)	39.2 (35.0-41.6)	41.7 (26.7-48.8)	44.6 (39.1-45.2)

### 3.5 Diagnostic utility of inflammatory indicators

**Figure 6:**
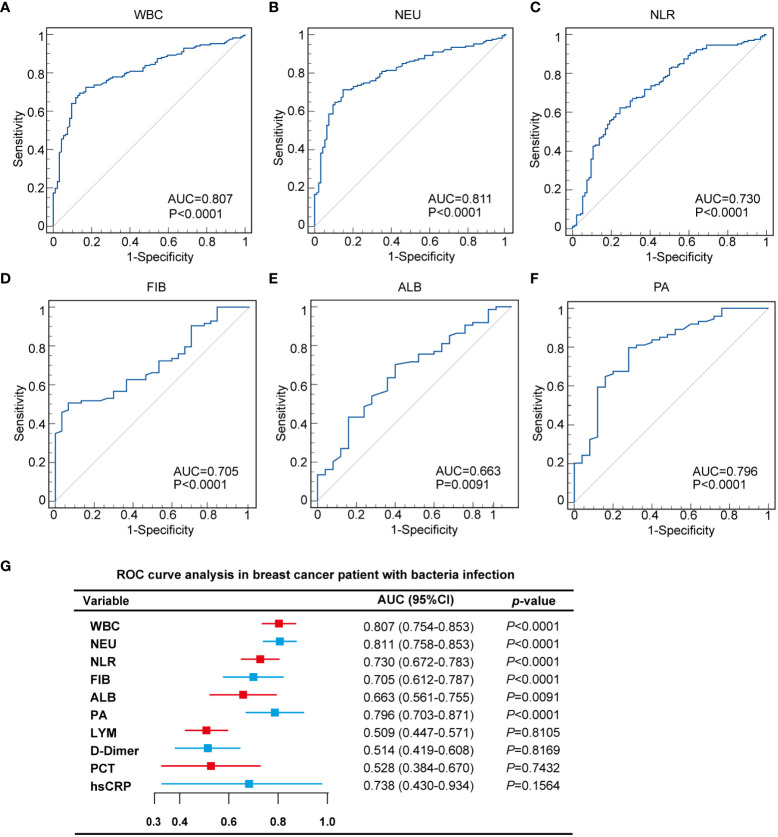
Diagnostic utility of inflammatory indicators in breast cancer patients with SSI. **(A)** ROC curves analyzed that the AUC of WBC was 0.807. **(B)** ROC curves analyzed that the AUC of NEU was 0.811. **(C)** ROC curves analyzed that the AUC of NLR was 0.730. **(D)** ROC curves analyzed that the AUC of FIB was 0.705. **(E)** ROC curves analyzed that the AUC of ALB was 0.663. **(F)** ROC curves analyzed that the AUC of PA was 0.796. **(G)** 95% CI and *p*-value of ROC curves.

We constructed ROC curves to evaluate the diagnostic utility of inflammatory indicators in breast cancer patients. The results showed that in the SSI group, WBC and NEU had discriminative power, with AUCs of 0.807 and 0.811, respectively, when compared with the control group (**Figures 6A, B**). For NLR, FIB, ALB and PA, the AUCs were 0.73, 0.705, 0.663 and 0.796, respectively (**Figures 6C-F**). The confidence interval (CI), sensitivity and specificity analysis of various inflammatory indicators are shown in **Figure 6G** and **Table 6**. Meanwhile, we constructed ROC curves for LYM, D-Dimer, PCT and hsCPR, however, their AUCs for these parameters were small and were not statistically significant (**Figures S3**, **Table S1**). We calculated cut-off values for WBC (8.57×109/L), NEU (6.48×109/L), NLR (5.27), FIB (4.13 g/L), ALB (40.7 g/L), and PA (0.214 mg/L). The cut-off values of NEU, NLR and FIB were higher than the normal range. The cut-off value of WBC was within the normal range, but close to the maximum reference value. The cut-off value of ALB was also within the normal range, but close to the minimum reference value. The cut-off value of PA was lower than the normal range. We think that these cut-off values could better help clinicians determine whether a breast cancer patient has SSI. More information relating to ROC curve analysis is shown in **Table 6**.

**Table 6:** The diagnostic performance comparison of WBC, NEU, NLR ALB and PA between SSI and control group in breast cancer patients.

Diagnostic performance	WBC	NEU	NLR	FIB	ALB	PA
AUC	0.807	0.811	0.730	0.705	0.663	0.796
95% CI	0.754-0.853	0.758-0.856	0.672-0.783	0.612-0.787	0.561-0.755	0.703-0.871
Cut-off value	8.57×10^9^/L	6.48×10^9^/L	5.27	4.13 g/L	40.7 g/L	0.214 g/L
Sensitivity	0.69	0.71	0.62	0.51	0.70	0.80
Specificity	0.86	0.85	0.75	0.93	0.60	0.72
PPV	0.89	0.89	0.81	0.93	0.84	0.89
NPV	0.39	0.38	0.47	0.60	0.61	0.47
Youden index	0.5563	0.5636	0.3781	0.4394	0.3027	0.5173
*p*-value	<0.0001	<0.0001	<0.0001	<0.0001	0.0091	<0.0001

### 3.6 Analysis of the drug resistance of pathogenic bacteria

**Figure 7:**
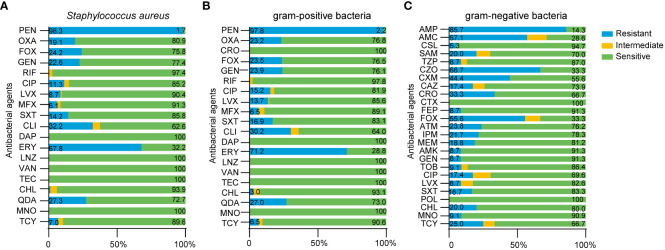
Drug resistance of pathogenic microorganism in breast cancer patients with SSI. **(A)** Antimicrobial susceptibility results of *Staphylococcus aureus*, **(B)** gram-positive bacteria, **(C)** gram-negative bacteria.

Finally, we analyzed the drug resistance of different type of bacteria. We found that the drug resistance rates of *Staphylococcus aureus* to oxacillin (OXA), ciprofloxacin (CIP) and levofloxacin (LVX) were 19.13%, 11.30% and 8.7%, while the drug resistance rates of gram-positive bacteria were 23.19%, 15.22% and 13.67%, respectively (**Figures 7A, B**). Because *Staphylococcus aureus* accounted for 81.5% of gram-positive bacteria, the resistance rate of gram-positive bacteria was not significantly different when compared to *Staphylococcus aureus*. Compared with gram-positive bacteria, gram-negative bacteria showed higher resistance to CRO (0 vs 33.33%), FOX (23.53 vs 55.56%), CHL (2.97 vs 20%), MNO (0 vs 9.09%) and TCY (6.52 vs 25%); furthermore, gram-negative bacteria showed lower resistance to GEN (23.91 vs 8.7%) (**Figures 7B, C**). Detailed information relating to the resistance rates of pathogenic microorganisms are given in **Tables 7** and **8**.

**Table 7:** Comparison of drug resistance of gram-positive bacteria and *Staphylococcus aureus* in breast cancer patients.

Antimicrobial agents	Drug resistance rate	
	gram-positive bacteria (%)	*Staphylococcus aureus* (%)
PEN (penicillin)	97.84	98.26
OXA (oxacillin)	23.19	19.13
FOX (cefoxitin)	23.53	24.24
GEN (gentamicin)	23.91	22.61
RIF (rifampin)	0	0
CIP (ciprofloxacin)	15.22	11.30
LVX (levofloxacin)	13.67	8.70
MFX (moxifloxacin)	6.52	6.09
SXT (Sulfamethoxazole Tablets)	16.91	14.16
CLI (clindamycin)	30.22	32.17
DAP (daptomycin)	0	0
ERY (erythromycin)	71.22	67.83
LNZ (linezolid)	0	0
VAN (vancomycin)	0	0
TEC (teicoplanin)	0	0
CHL (chloramphenicol)	2.97	1.22
QDA (Quinuputin/Dafuputin)	27.03	27.27
MNO (minocycline)	0	0
TCY (tetracycline)	6.52	6.96

**Table 8:** Compare of drug resistance of gram-positive bacteria and gram-negative bacteria in breast cancer patients.

Antimicrobial agents	Drug resistance rate	
	gram-positive bacteria (%)	gram-negative bacteria (%)
CRO (ceftriaxone)	0	33.33
FOX (cefoxitin)	23.53	55.56
GEN (penicillin)	23.91	8.70
CIP (ciprofloxacin)	15.22	17.39
LVX (levofloxacin)	13.67	8.70
SXT (Sulfamethoxazole Tablets)	16.91	16.67
CHL (chloramphenicol)	2.97	20
MNO (minocycline)	0	9.09
TCY (tetracycline)	6.52	25

## 4 Discussion

Cancer is a leading cause of death and a significant barrier to longer life expectancy for every country in the world (Bray et al., 2021). Female breast cancer is the main cause of global cancer incidence and the fifth leading cause of cancer mortality worldwide, with 685,000 deaths annually (Sung et al., 2021). Surgery is still the standard treatment for breast cancer (O'Connor et al., 2020). However, SSI has become an important factor that affects the prognosis of patients with breast cancer. SSI can increase the financial burden to patients, delay postoperative radiotherapy, increase metastatic relapse and reduced survival exist rates (Zukowska and Zukowski, 2022). In our analysis, we found that the use of antibiotic prophylaxis could not reduce the likelihood of SSI; this finding was in line with previous studies (Prudencio et al., 2020). The relevant data of patients with a local recurrence were not included in this paper. Therefore, we did not analyze whether SSI was associated with a local recurrence, which suggests that we need to further track the patients’ disease course and analyze the relationship between SSI and breast cancer recurrence in the future.

The levels of many inflammatory indicators change when SSI occurs. Therefore, it is important to find effective indicators to ensure that SSI is diagnosed and treated in a timely. In this study, we analyzed a range of inflammatory markers including blood-related indicators (WBC, NEU, LYM, NLR, D-Dimer, FIB, ALB, PA) and classical inflammatory markers (PCT and hsCRP). WBC, NEU and NLR all increased significantly in SSI (**Figure 2A-C**, **3A-C**); these findings were consistent with previous research (Huh et al., 2019; Inose et al., 2019). Blood clotting related indicators were also closely associated with infection (King et al., 2014). Consistent with these findings, we found that FIB was also significantly increased in patients with SSI (**Figure 3D**). Previous studies have found that patients with low PA and ALB levels prior to surgery have an increased risk of SSI (Salvetti et al., 2018; Mentor et al., 2020); our present findings concurred with these previous data (**Figure 3E, F**). PCT and CRP have received increasing levels of attention with regards to the diagnosis of infection (Tang et al., 2018; Giannini et al., 2019). However, the amount of data related to PCT and CRP in these cases was insufficient to allow us to analyze their correlation with SSI; this requires further investigation. In addition to the analysis of inflammatory indicators, we also analyzed the clinical characteristics of our patients. We found that BMI was correlated with the SSI in breast cancer patients (**Table 1**). Other research also shown that obesity may increase the risk of breast cancer and that losing weight can reduce the risk of breast cancer (Lee et al., 2019; Crafts et al., 2022). This highlights the fact that exercise and weight control are important for the prevention of cancer.

In order to further explore the changes of inflammatory indicators caused by different types of bacterial infections in SSI, we first classified the infectious bacteria. Growing evidence shows that *Staphylococcus aureus*, including MRSA, is the main cause of SSI (Saadatian-Elahi et al., 2008; Patel et al., 2017). In our study, we found that the infection rate caused by *Staphylococcus aureus* reached as high as 70.41% in breast cancer patients with SSI (**Figure 4A**). In addition, our analysis also showed that WBC and NEU increased more significantly with *Staphylococcus aureus* infection than with any other bacterial infection (**Figures 5A, B**). Consistent with these results, the AUCs for WBC and NEU were both > 0.8, thus indicating that they had superior diagnostic value for SSI (**Figures 6A, B**). Finally, we analyzed the drug sensitivity of *Staphylococcus aureus*, gram-positive bacteria and gram-negative bacteria (**Figures 7A-C**), hoping that our results could provide reference guidelines for the timely and accurate drug treatment of SSI in breast cancer patients.

With the continuous development of detection technology, an increasing number of inflammatory markers have been developed and utilized. The levels of cytokines, especially IL-6 and IL-10, usually increase during bacterial infection and are considered to represent early biomarkers to assist in the diagnosis of bacterial infections (Ye et al., 2020; Zhu et al., 2022). Acute-phase reactant serum amyloid A (A-SAA) is used in clinical laboratories as an indicator of inflammation and is more conclusive than the detection of CRP in patients with viral infections, and severe acute pancreatitis (Ye et al., 2020; Zhu et al., 2021). Human neutrophil lipocalin (HNL) is a novel inflammatory marker; the diagnostic efficacy of HNL is not affected by the site of infection or by the pathogenic bacterial species involved (Fang et al., 2020). Heparin-binding protein (HBP) is another novel inflammatory factor released from neutrophils (Wang et al., 2022). HBP can directly kill bacteria and enhance bacterial clearance by attracting immune cells to the site of infection and is considered to represent a novel and valuable biomarker for infectious disease (Katsaros et al., 2022; Xue et al., 2022). This study revealed that WBC, NEU, NLR, FIB and PA have good predictive value for identifying SSI in breast cancer patients. Meanwhile, the test of antimicrobial susceptibility could be helpful in the treatment of SSI caused by different type of bacteria. In order to improve diagnosis and treatment times of SSI, we need to expand the number of specimens and increase new inflammatory markers in the future research.

## Data availability statement

The raw data supporting the conclusions of this article will be made available by the authors, without undue reservation.

## Ethics statement

The studies involving humans were approved by Ethics Committee of the Shandong Cancer Hospital and Institute. The studies were conducted in accordance with the local legislation and institutional requirements. The participants provided their written informed consent to participate in this study. Written informed consent was obtained from the individual(s) for the publication of any potentially identifiable images or data included in this article.

## Author contributions

DL: Conceptualization, Writing – original draft. SD: Writing – original draft. JL: Writing – original draft. XL: Writing – original draft. KR: Writing – review & editing. LL: Writing – review & editing. WS: Conceptualization, Formal Analysis, Writing – original draft, Writing – review & editing.
