# Supplementary material for: Diagnostic value of inflammatory indicators for surgical site infection in patients with breast cancer
Source: Front Cell Infect Microbiol. 2023 Oct 25;13:1286313. doi: 10.3389/fcimb.2023.1286313 (PMC10634473; doi:10.3389/fcimb.2023.1286313)
Supplement: Supplementary file 1 [file Table_1.docx]

**Supplementary material**


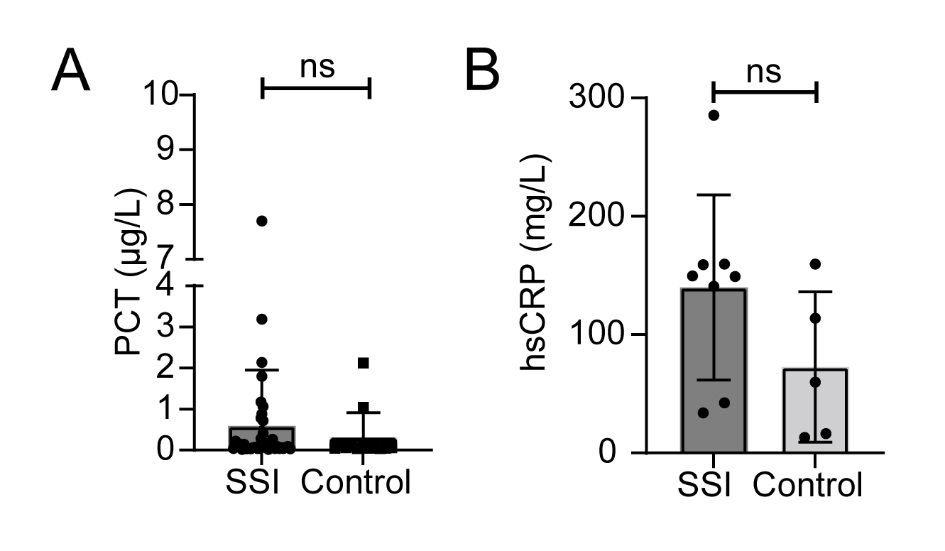


**Supplementary Figure 1** Changes of PCT **(A)** and hsCRP **(B)** in SSI and control groups.


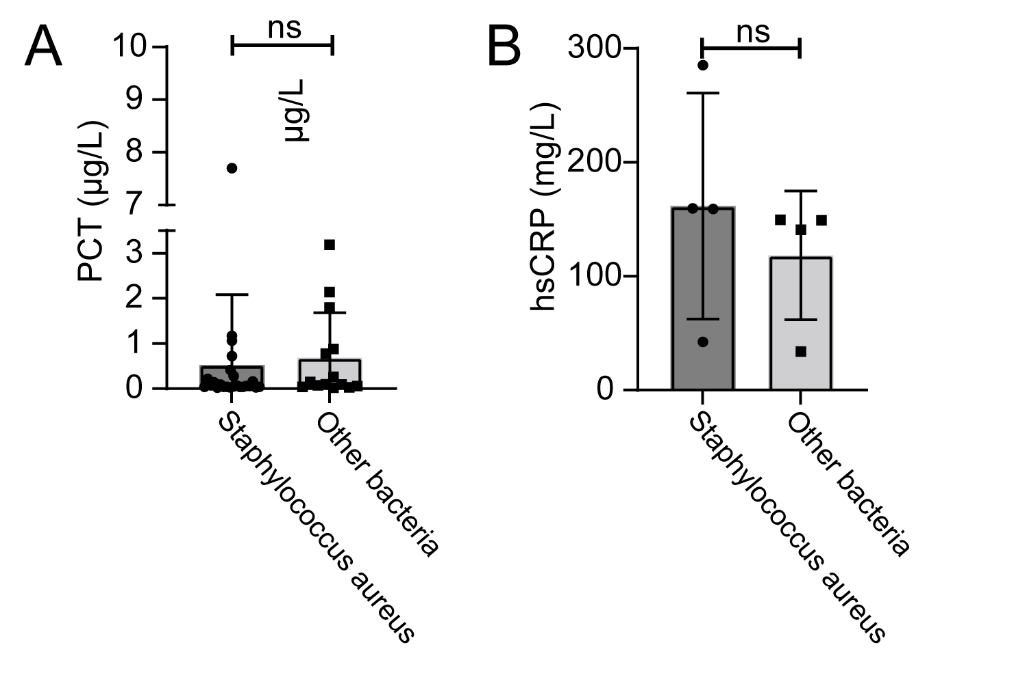


**Supplementary Figure 2** Changes of PCT **(A)** and (hsCRP) after infection with *Staphylococcus aureus* and other bacteria.


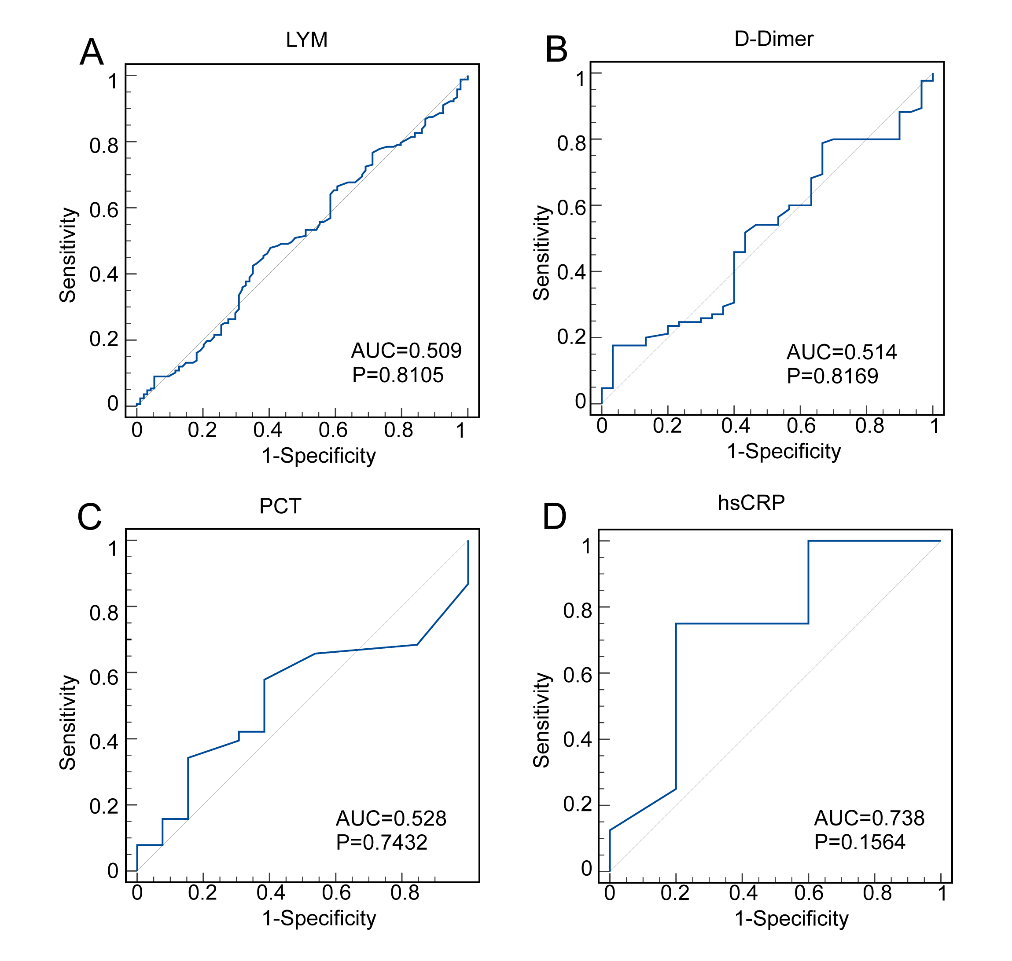


**Supplementary Figure 3** Diagnostic utility of inflammatory indicators in breast cancer patients with SSI. **(A)** ROC curves analyzed that the AUC of LYM was 0.509. **(B)** ROC curves analyzed that the AUC of D-Dimer was 0.514. **(C)** ROC curves analyzed that the AUC of PCT was 0.528. **(D)** ROC curves analyzed that the AUC of hsCRP was 0.738.

**Supplementary Table 1**: The diagnostic performance comparison of LYM, D-Dimer, PCT and hsCRP between SSI and control group in breast cancer patients.

| **Diagnostic performance** | **LYM** | **D-Dimer** | **PCT** | **hsCRP** |
| --- | --- | --- | --- | --- |
| AUC | 0.509 | 0.514 | 0.528 | 0.738 |
| 95% CI | 0.447-0.571 | 0.419-0.608 | 0.384-0.670 | 0.430-0.934 |
| Cut-off value | 1.3×10^9^/L | 2.12 mg/L | 0.06 μg/L | 113.9 mg/L |
| Sensitivity | 0.48 | 0.18 | 0.58 | 0.75 |
| Specificity | 0.60 | 0.97 | 0.62 | 0.80 |
| PPV | 0.67 | 0.94 | 0.78 | 0.75 |
| NPV | 0.61 | 0.70 | 0.68 | 0.40 |
| Youden index | 0.0748 | 0.1431 | 0.1943 | 0.55 |
| *p*-value | 0.8105 | 0.8169 | 0.7432 | 0.1564 |
